# Retrospective clinical study of renin-angiotensin system blockers in lung cancer patients with hypertension

**DOI:** 10.7717/peerj.8188

**Published:** 2019-12-10

**Authors:** Jie Wei, Zhiyang Zhou, Zhijie Xu, Shuangshuang Zeng, Xi Chen, Xiang Wang, Wanli Liu, Min Liu, Zhicheng Gong, Yuanliang Yan

**Affiliations:** 1 Department of Pharmacy, Xiangya Hospital, Central South University, Changsha, Hunan, China; 2 Institute for Rational and Safe Medication Practices, National Clinical Research Center for Geriatric Disorders, Xiangya Hospital, Central South University, Changsha, Hunan, China; 3 Department of Breast Surgery, Xiangya Hospital, Central South University, Changsha, Hunan, China; 4 Department of Pathology, Xiangya Hospital, Central South University, Changsha, Hunan, China

**Keywords:** Lung cancer, Hypertension, Renin-angiotensin system blockers, Clinical analysis

## Abstract

**Purpose:**

Renin-angiotensin system blockers (RASBs), which include angiotensin-converting enzyme inhibitors (ACEIs) and angiotensin-2 receptor 1 blockers (ARBs), have been reported to be associated with lung cancer metastasis, radiotherapy and chemotherapy. Until now, very limited clinical data for RASBs’ diagnostic and prognostic effects has existed for lung cancer chemotherapy in Chinese patients.

**Methods:**

There were a total of 678 lung cancer patients with hypertension, of which 461 (68%) were in the non-RASBs group and 217 (32%) were in the RASBs group. Patients’ gender, age, smoking status, histologic differentiation, tumor size, pathological grade, lymph node metastasis, pathological stage and progression-free survival (PFS) were retrospectively analyzed between these two groups. The clinical effects of ACEIs and ARBs in lung cancer patients were compared via t tests, and *χ*^2^ test, and potential prognostic factors for progression-free survival (PFS) were evaluated by Kaplan–Meier analysis.

**Results:**

Significant differences were observed in lymph node metastasis between the RASBs and non-RASBs groups. The RASBs group (62.8% vs 71.7%, *p* = 0.037) and ARBs group (60.0% vs 71.7%, *p* = 0.030) had lower lymph node metastasis, and patients with RASBs had a lower pathological stage than those in non-RASBs groups (67.1% vs 77.4%, *p* = 0.044 ). The PFS of the RASBs (10.7 vs. 6.7 months, *p* = 0.040) and ACEIs (12.9 vs 6.7 months, *p* = 0.021) groups were longer than that of the non-RASBs group, while no statistical difference was shown between the ACEIs and ARBs groups. Moreover, the significant results of PFS were further confirmed in pathological stage III–IV patients. In the non-RASB group, 55% of patients took calcium channel blockers (CCBs), and the ACEIs group have a significantly longer PFS compared to the non-CCBs group (6.4 vs 12.9 months, *p* = 0.036).

**Conclusion:**

In this study, we showed that the use of RASBs is a positive factor for pathological stage and prognosis of lung cancer patients. Therefore, it is necessary to actively evaluate medical history, especially the use of anti-hypertension medication, in patients with lung cancer and reflect medical history in the treatment and management plans of these patients.

## Introduction

Lung cancer is one of the most common malignant tumors and causes more cancer deaths per year than the next three cancers combined in China. Despite remarkable advances in targeted therapy, the morbidity and mortality of lung cancer still have not significantly improved worldwide (Lu, Yu & Yang, 2019; Wang et al., 2019). Statistics from GLOBOCAN 2012, produced by the International Agency for Research on Cancer (IARC), indicated that approximately 1.8 million new cases of and approximately 1.6 million deaths from lung cancer each year (Torre et al., 2015). The survival rates for lung cancer are abysmal, which are in stark contrast to the high survival rates of breast, colon and prostate cancer (Bach et al., 2012). Presently, clinical treatment of lung cancer is generally based on pathological type, stage of tumors, and comprehensive assessment of the overall state of the patients to select appropriate methods to positively treat, improve symptoms, and prolong survival. The main treatment methods include minimally invasive surgery, precision radiotherapy, molecular targeted medicine therapy, Chinese medicine therapy, immunotherapy and psychotherapy. Although surgery, new molecular targeted medicine therapy and precision radiotherapy technology have greatly improved therapeutic effects in lung cancer patients, contrary to most Western countries, lung cancer incidence rates in China are still increasing, and the treatment of lung cancer remains a clinical problem to be solved (Lu, Yu & Yang, 2019).

In addition to long-term smoking, comorbidities are considered as important factors that affect treatment decisions, treatment processes and the prognoses of lung cancer patients and are the main cause of drug resistance and death in patients with lung cancer. Cancer-related comorbidities include hypertension, diabetes, obesity, coronary atherosclerotic heart diseases, and chronic obstructive pulmonary diseases. Hypertension is one of the prominent comorbidities in cancer and affects the survival of cancer patients (Nakaya et al., 2016; Shen et al., 2016). A retrospective analysis revealed that blood pressure was an independent unfavorable factor for tumors (Rhodes et al., 2009). For breast cancer patients, the higher the blood pressure, the higher the likelihood of developing a tumor (Han et al., 2017). Recently, studies have shown that anti-hypertensive drugs have potential anti-tumor effects. Compared with no use of anti-hypertensive drugs, anti-hypertensive drugs such as thiazide diuretics could substantially increase the risk of squamous cell carcinoma (Pedersen et al., 2018). The use of calcium channel blockers (CCBs) in lung cancer patients with hypertension increased the risk of death, while renin-angiotensin system blockers (RASBs) improved overall survival in the Caucasian population(Menter et al., 2017; Rotshild et al., 2018). However, beta-blockers seem to have no effect on the prognosis of breast, lung, and colorectal cancer patients (Musselman et al., 2018; Yang et al., 2017). These outcomes indicate that the effects of anti-hypertensive drugs vary, especially in different cancers, based on specific pharmacologic action.

RASBs include angiotensin-converting enzyme inhibitors (ACEIs) and angiotensin II receptor blockers (ARBs), which are used as the initial therapeutic drugs in hypertension treatment guidelines throughout the world. For their potential for anticancer intervention, RASBs have received considerable attention. The original report on the anticancer effect of ACEIs originated from a retrospective cohort in 1998 (Lever et al., 1998). Subsequently, a large number of studies showed a clear protective association between RASBs and lung cancer in Asian and Caucasian populations (Bhaskaran et al., 2012; Huang et al., 2011; Pinter & Jain, 2017; Rao et al., 2013; Wang et al., 2015). However, Bangalore et al. (2011) showed that anti-hypertensive therapy with a combination of ACEIs and ARBs increased the risk of cancer. A meta-analysis that used large-scale clinical trial data suggested that users of ARBs could have an increased risk of lung cancer (Sipahi et al., 2010). Double-blind clinical trials, including 15 large parallel long-term multicenter studies, refuted the relative increase in cancer risk (ARBT Collaboration, 2011). While a population-based cohort study showed that patients with ACEIs over 5 years have a significant risk of lung cancer (Hicks et al., 2018). In addition, these studies have limitations, such as being prone to residual confounding factors (Cronin-Fenton, 2018).

The impacts of RASBs on lung cancer remains debated. Regarding the impact of anti-hypertensive drugs, RASBs have been found to be controversial in the risk and OS of lung cancer in different racial groups. However, patients’ clinical stage and pathological grades were not included. Considering the lack of clinical data and ethnic differences, the present study was designed to comprehensively compare both the pathological and prognostic effect of RASBs, as well as ACEIs and ARBs, in Chinese lung cancer patients.

## Material and Methods

### Patients

**Figure 1:**
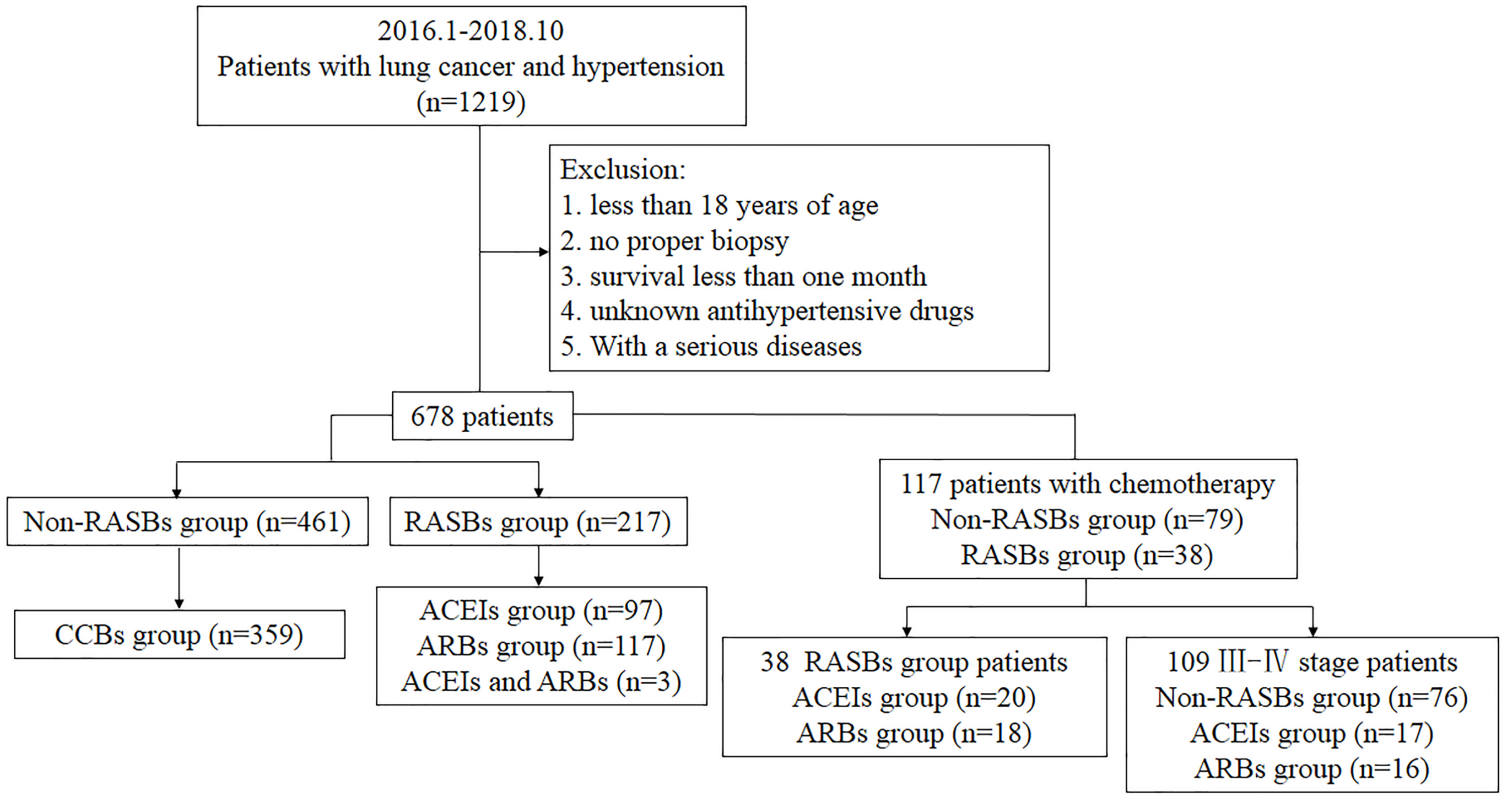
Flow chart of recruiting patients.

This study was conducted among hospitalized patients in Xiangya Hospital. A total of 1219 Chinese patients who were diagnosed with lung cancer and hypertension from January 2016 to October 2018 were retrospectively recruited from the Intravenous Prescription Early Warning and Assessment System, Intravenous Infusion Safety Evaluation Center of Hunan Province, China. We excluded the patients with no proper biopsy, those younger than 18 years of age, those who had a survival time of less than one month after the diagnosis of cancer, those on unknown anti-hypertensive drugs, and those with serious diseases. All patients took anti-hypertensive drugs at least six months before the first diagnosis of lung cancer. Finally, 678 patients were enrolled. According to whether they were taking RASBs, patients were divided into RASBs (*n* = 217) and non-RASBs groups (*n* = 461) (including *β*-adrenoceptor blockers, *α*-adrenoceptor blockers, CCBs, vasodilators, centrally acting antihypertensives, diuretics, and ganglion blockers) (Fig. 1). The RASBs group was further divided into ACEIs and ARBs groups.

### Clinical data

This study was reviewed and approved by the Ethical Committee of Xiangya Hospital of Central South University (Approval No. 201906139). Clinical patient indicators were collected including gender, age (Myneni et al., 2013), smoking status, histologic differentiation, tumor size (Cheung et al., 2014), lymph node metastasis, pathological grade and pathological stage. Histologic differentiation and TNM categories were graded on the basis of the World Health Organization (WHO) and the 8th edition of the American Joint Committee on Cancer (AJCC) (Morrow, 2017). Subgroup analyses were further performed in lung cancer patients who received regular platinum-based chemotherapy (*n* = 117), as well as in stage III-IV patients who received platinum-based chemotherapy (*n* = 107) (Fig.  1). Progression-free survival (PFS) was defined as the time from the onset of anti-cancer treatment to the date of disease progression or death from any cause. Follow-up time was defined as the time from the date of diagnosis to progression or the last follow-up. For the evaluation of tumor response, CT, MRI or SPECT scans were reviewed by specialized radiologists and response was determined by Response Evaluation Criteria in Solid Tumors (RECIST) (Eisenhauer et al., 2009).

### Statistical analyses

All statistical analyses were performed using SPSS 18 software (SPSS Inc, Chicago, USA) and GraphPad Prism 6 (GraphPad Software, Inc., La Jolla, CA, USA). Measurable data were expressed as the mean and standard deviation (SD). Differences in patient and tumor characteristics between groups were compared using Pearson’s chi-square or Fisher’s exact tests. The Kaplan–Meier method was used to predict the PFS of cancer patients with or without RASBs after chemotherapy, and outcomes between groups were compared with the log-rank statistic. All statistical tests were 2-sided, and P values less than 0.05 were considered statistically significant.

## Results

### Clinicopathological characteristics between RASBs and non-RASBs

**Figure 2:**
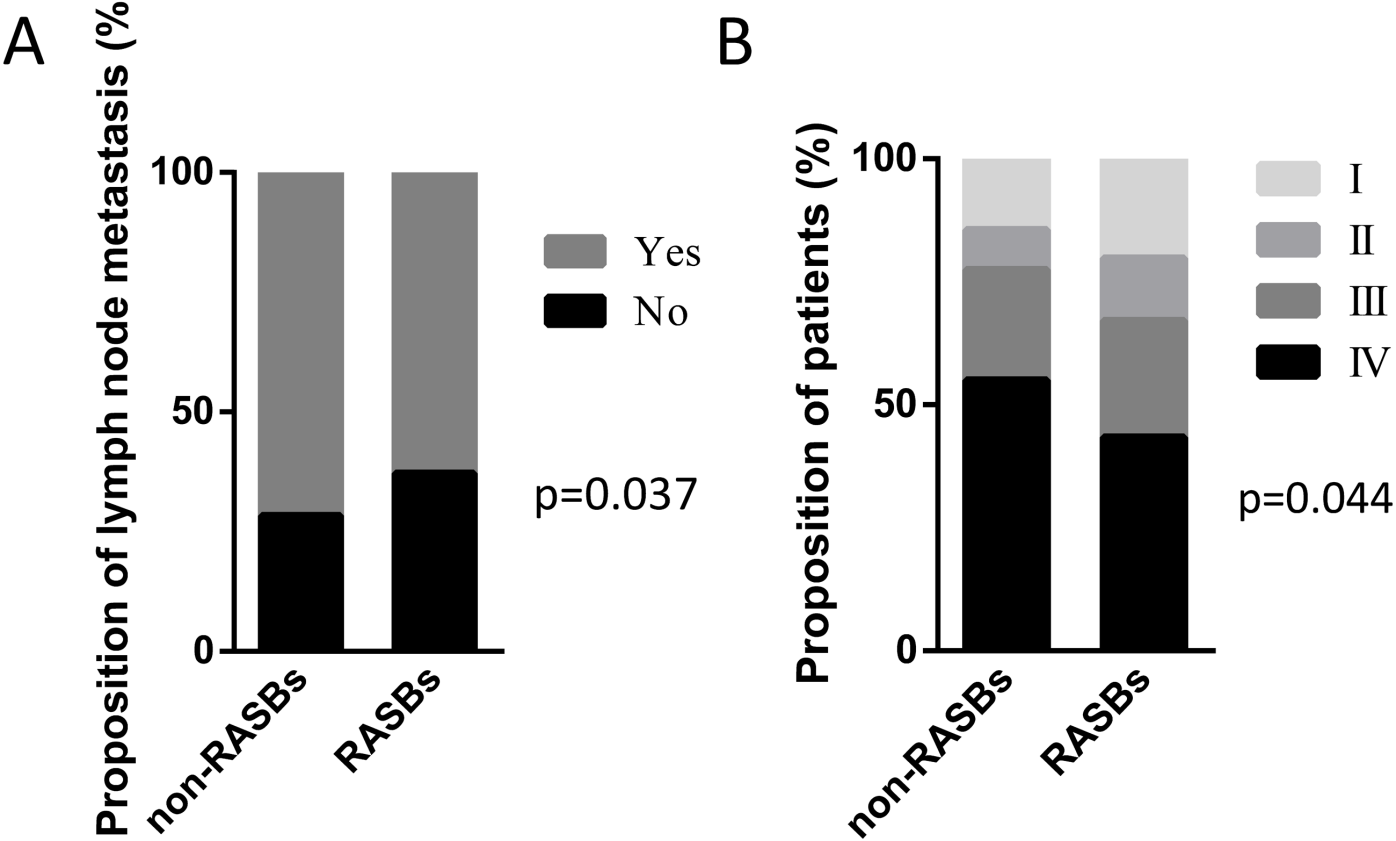
Graphical representations of the proportion of lymph node metastasis and pathological stage between the non-RASBs group and the RASBs group. (A) Lymph node metastasis; (B) pathological stage; the statistical significance for difference of means is shown (*P* values, *χ*^2^ test).

The cohort included 678 lung cancer patients with hypertension who received RASBs (*n* = 461, 68%) or non-RASBs (*n* = 217, 32%), respectively. The clinicopathological characteristics in 678 lung cancer patients are described in Table 1. Compared with the non-RASBs group, the RASBs group had a significantly lower lymph node metastasis rate (62.8% vs 71.7%, *p* = 0.037) and advanced pathological stage (67.1% vs 77.4%, *p* = 0.044) (Fig. 2). However, there were no significant differences in other parameters such as gender, age, smoking status, histology, tumor size and pathological grade between these two groups (*p* > 0.05).

**Table 1:** Clinicopathological characteristics in 678 lung cancer patients. RASBs, renin-angiotensin system blockers; ACEIs, angiotensin-converting enzyme inhibitors; ARBs, angiotensin-2 receptor 1 blockers; SD, standard deviation; NSCLC, non-small cell lung cancer; SCLC, small cell lung cancer.

Characteristic	Non-RASBs, *n* = 461	RASBs					
		Total, *n* = 217	*P*-value^b^	ACEIs^a^, *n* = 97	*P*-value^b^	ARBs^a^, *n* = 117	*P*-value^b^
Gender, *n* (%)							
Male	314 (68.1)	144 (66.4)	0.649	63 (64.9)	0.545	78 (66.7)	0.765
Female	147 (31.9)	73 (33.6)		34 (35.1)		39 (33.3)	
Age, years, *n* (%)							
Mean ± SD	64.71 ± 8.10	63.52 ± 7.49	0.183	64.62 ± 8.39	0.914	64.06 ± 8.07	0.328
≤60	125 (27.1)	62 (28.6)	0.692	34 (35.1)	0.115	27 (23.1)	0.376
>60	336 (72.9)	155 (71.4)		63 (64.9)		90 (76.9)	
Smoking status, *n* (%)							
Never	181 (39.3)	96 (44.2)	0.219	40 (41.2)	0.718	56 (47.9)	0.091
Ever	280 (60.7)	121 (55.8)		57 (58.8)		61 (52.1)	
Histology, *n* (%)							
NSCLC	381 (82.6)	182 (83.9)	0.872	80 (82.5)	0.949	99 (84.6)	0.858
Adenocarcinoma	263 (57.0)	119 (54.8)		53 (54.6)		64 (54.7)	
Squamous	98 (21.3)	47 (21.7)		21 (21.6)		26 (22.2)	
SCLC	64 (13.9)	27 (12.4)		13 (13.4)		14 (12.0)	
Others	16 (3.5)	8 (3.7)		4 (4.1)		4 (3.4)	
Tumor size, *n* (%)							
Mean ± SD	41.54 ± 22.30	38.89 ± 20.39	0.177	39.49 ± 20.71	0.447	38.57 ± 20.33	0.236
≤3 cm	149 (32.3)	73 (33.6)	0.621	33 (34.0)	0.720	39 (33.3)	0.716
>3 cm	237 (51.4)	106 (48.9)		48 (49.5)		57 (48.7)	
Unknown	75 (16.3)	38 (17.5)		16 (16.5)		21 (18.0)	
Pathological grade, *n* (%)							
Well	51 (11.1)	23 (10.6)	0.900	12 (12.4)	0.562	10 (8.5)	0.910
Moderately	132 (28.6)	60 (27.7)		30 (30.9)		29 (24.8)	
Poorly	98 (21.3)	40 (18.4)		16 (16.5)		23 (19.7)	
Unknown	180 (39.0)	94 (43.3)		39 (40.2)		55(47.0)	
Aspirin							
Yes	18 (3.9)	16 (7.4)	0.054	5 (5.2)	0.778	11 (9.4)	0.015^*^
No	443 (96.1)	201 (92.6)		92 (94.8)		106 (90.6)	

**Notes.**

a Patients who took ACEIs and ARBs were excluded.

b Each group was separately compared with the Non-RASBs group.

* *P* < 0.05.

### Clinicopathological characteristics between ACEIs and ARBs

**Figure 3:**
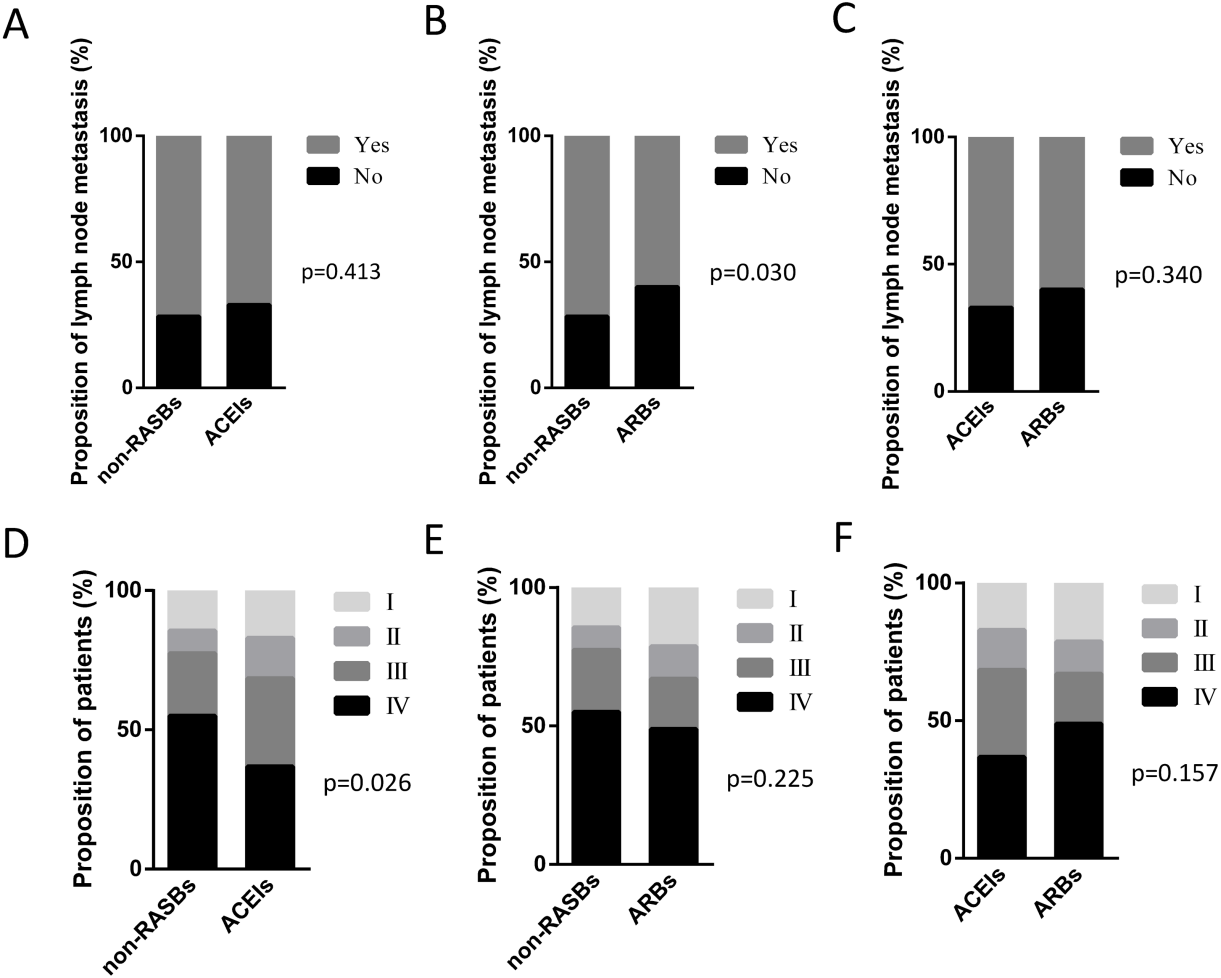
Graphical representations of the proportion of lymph node metastasis and pathological stage between the non-RASBs group and the ACEIs or ARBs group. Comparison of lymph node metastasis between (A) the non-RASBs group and ACEIs group; (B) the non-RASBs group and ARBs group; (C) the ACEIs group and ARBs group. Comparison of the pathological stage between (D) the non-RASBs group and ACEIs group; (E) the non-RASBs group and ARBs group; (F) the ACEIs group and ARBs group. The statistical significance for difference of means is shown (*P* values, *χ*^2^ test or Fisher’s exact test).

Among the 217 patients with RASBs, 97 cases received ACEIs and 117 cases received ARBs. Three patients who received both ACEIs and ARBs were excluded for further analyses. We analyzed the lymph node metastasis and pathological stage between the non-RASBs, ACEIs and ARBs groups. Compared with the non-RASBs group, the lymph node metastasis rate was significantly statistically lower in the ARBs group (60.0% vs 71.7%, *p* = 0.030) (Fig. 3B). However, the lymph node metastasis rate was not statistically significant between the ACEIs and non-RASBs or ARBs groups (Figs. 3A, 3C). For pathological stage, the ACEIs group had significantly more advanced stage patients than the non-RASBs group (68.4% vs 77.4%, *p* = 0.026) (Fig. 3D), while there was no significant difference between the ARBs and non-RASBs or ACEIs groups (Figs. 3E, 3F).

### PFS of lung cancer patients with chemotherapy

**Figure 4:**
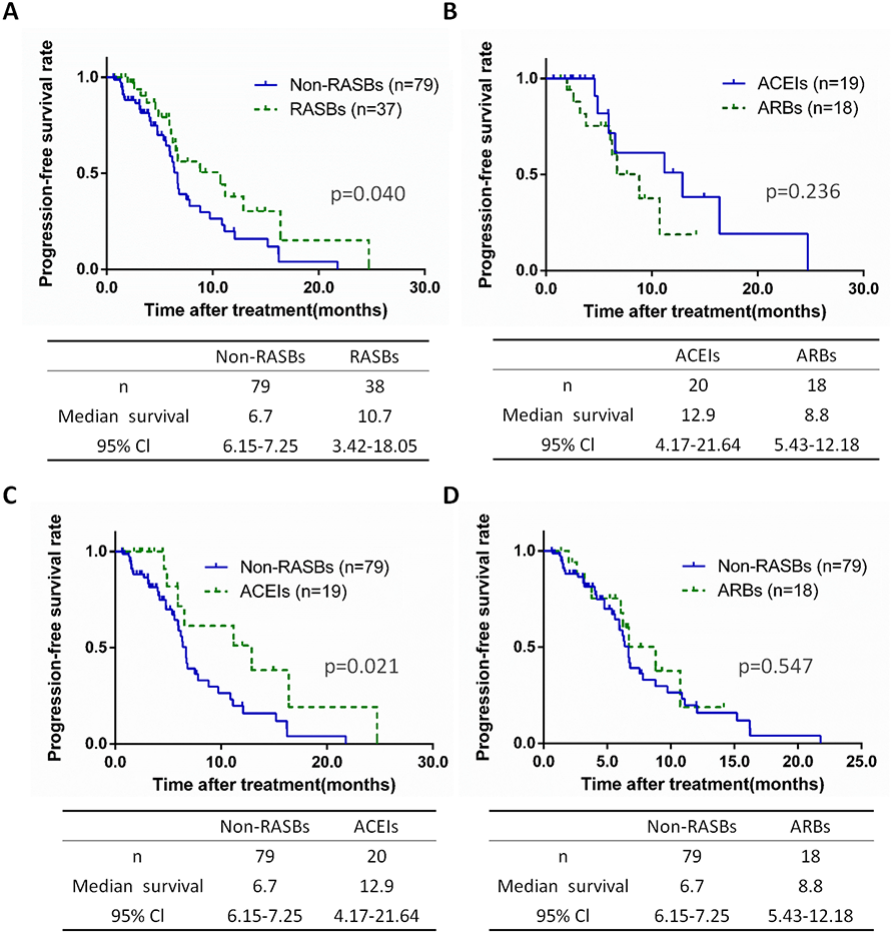
Kaplan–Meier curves for PFS in 117 lung cancer patients receiving chemotherapy. Kaplan–Meier method analysis of PFS between (A) the non-RASBs group and RASBs group; (B) the ACEIs group and ARBs group; (C) the non-RASBs group and ACEIs group; (D) the non-RASBs group and ARBs group. The statistical significance for difference of means is shown.

A total of 117 patients received chemotherapy, including 79 non-RASBs cases (67.5%) and 38 RASBs cases (32.5%) (Table 2). The PFS of each group were assessed and significant differences were calculated between the non-RASBs and RASBs, ACEIs and ARBs, non-RASBs and ACEIs, and non-RASBs and ARBs groups. Compared to the non-RASBs group, the PFS of the RASBs group was 4.0 months longer in lung cancer patients with a significant difference (10.7 vs. 6.7 months, *p* = 0.040) (Fig. 4A). For subgroup analyses, the PFS of the ACEIs group was significantly longer, approximately 6.2 months longer than that of the non-RASBs group (12.9 vs 6.7, *p* = 0.021) (Fig. 4C). However, Kaplan–Meier analysis revealed no significant difference between the ACEIs and ARBs groups, as well as between the non-RASBs and ARBs groups (*p* > 0.05) (Figs. 4B, 4D).

**Table 2:** Clinicopathological characteristics in 117 lung cancer patients with platinum- based chemotherapy. RASBs renin-angiotensin system blockers; ACEIs angiotensin-converting enzyme inhibitors; ARBs angiotensin-2 receptor 1 blockers; SD standard deviation; NSCLC non-small cell lung cancer; SCLC small cell lung cancer.

Characteristic	Non- RASBs, *n* = 79	RASBs					
		Total, *n* = 38	*P*-value^a^	ACEIs, *n* = 20	*P*-value^a^	ARBs, *n* = 18	*P*-value^a^
Gender, *n* (%)							
Male	62 (78.5)	26 (68.4)	0.238	13 (65.0)	0.335	13 (72.2)	0.795
Female	17 (21.5)	12 (31.6)		7 (35.0)		5 (27.8)	
Age, years, *n* (%)							
Mean ± SD	62.96 ± 8.02	61.08 ± 7.89	0.995	61.35 ± 6.60	0.439	60.78 ± 9.31	0.425
≤60	27 (34.2)	17 (44.7)	0.269	10 (50.0)	0.191	7 (38.9)	0.705
>60	52 (65.8)	21 (55.3)		10 (50.0)		11 (61.1)	
Smoking status, *n* (%)							
Never	26 (32.9)	18 (47.4)	0.131	9 (45.0)	0.312	9 (50.0)	0.173
Ever	53 (67.1)	20 (52.6)		11 (55.0)		9 (50.0)	
Histology, *n* (%)							
NSCLC	60 (75.9)	31 (81.6)	0.567	16 (80.0)	0.766	15 (83.3)	0.543
Adenocarcinoma	43 (54.4)	19 (50.0)		11 (55.0)		8 (44.4)	
Squamous	17 (21.5)	12 (31.6)		5 (25.0)		7 (38.9)	
SCLC	18 (22.8)	7 (18.4)		4 (20.0)		3 (16.7)	
Others	1 (1.3)	0 (0)		0 (0)		0 (0)	
Tumor size, *n* (%)							
Mean ± SD	45.95 ± 19.39	49.55 ± 21.27	0.372	49.61 ± 17.99	0.797	49.47 ± 25.33	0.094
≤3 cm	18 (22.8)	6 (15.8)	0.482	2 (10.0)	0.196	4 (22.2)	0.849
>3 cm	56 (70.9)	27 (71.0)		16 (80.0)		11 (61.1)	
Unknown	5 (6.3)	5 (13.2)		2 (10.0)		3 (16.7)	
Pathological grade, *n* (%)							
Well	2 (2.5)	3 (7.9)	0.305	3 (15.0)	0.107	0 (0)	0.690
Moderately	14 (17.7)	7 (18.4)		4 (20.0)		3 (16.7)	
Poorly	29 (36.7)	10 (26.3)		5 (25.0)		5 (27.8)	
Unknown	34 (43.1)	18 (47.4)		8 (40.0)		10 (55.5)	

**Notes.**

a Each group was separately compared with the Non-RASBs group.

### PFS of advanced stage lung cancer patients with chemotherapy

**Figure 5:**
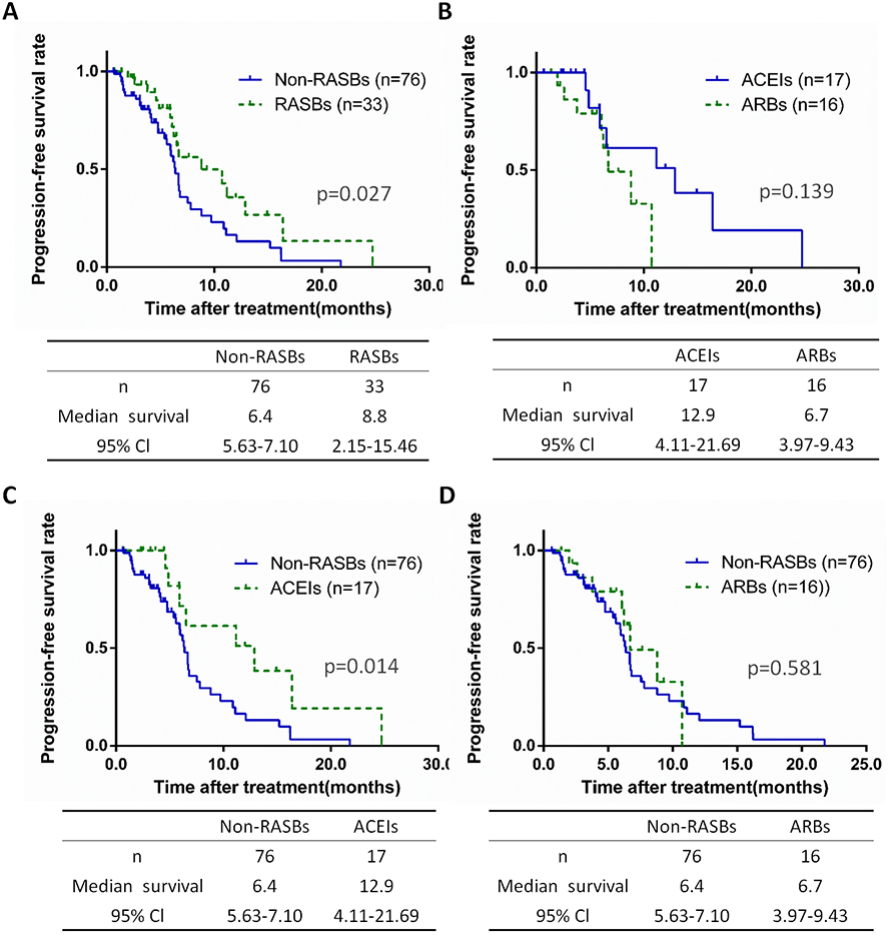
Kaplan-Meier curves for PFS in 109 stage III–IV lung cancer patients receiving chemotherapy. Kaplan-Meier method analysis of PFS between (A) the non-RASBs group and RASBs group; (B) the ACEIs group and ARBs group; (C) the non-RASBs group and ACEIs group; (D) the non-RASBs group and ARBs group. The statistical significance for difference of means is shown.

In 109 patients diagnosed as stage III-IV undergoing chemotherapy, 69.7% cases received non-RASBs (*n* = 76) and 30.3% received RASBs (*n* = 33), while RASBs contained 17 ACEIs (15.6%) and 16 ARBs (14.7%) cases. We further calculated the PFS of lung cancer at an advanced stage, based on non-RASBs, RASBs and subgroups. Similar results were found in stage III-IV lung cancer patients as stages I-IV. The RASBs group have a significantly longer PFS compared to the non-RASBs group (8.8 vs 6.4, *p* = 0.027), but no significant difference was found between the ACEIs and ARBs groups (Figs. 5A, 5B). In subgroup analyses, the results showed that the ACEIs group had a significantly longer PFS (12.9 vs 6.4, *p* = 0.014), while ARBs had no significant differences compared to the non-RASBs group (Figs. 5C, 5D).

### Prognostic analysis of CCBs group

**Figure 6:**
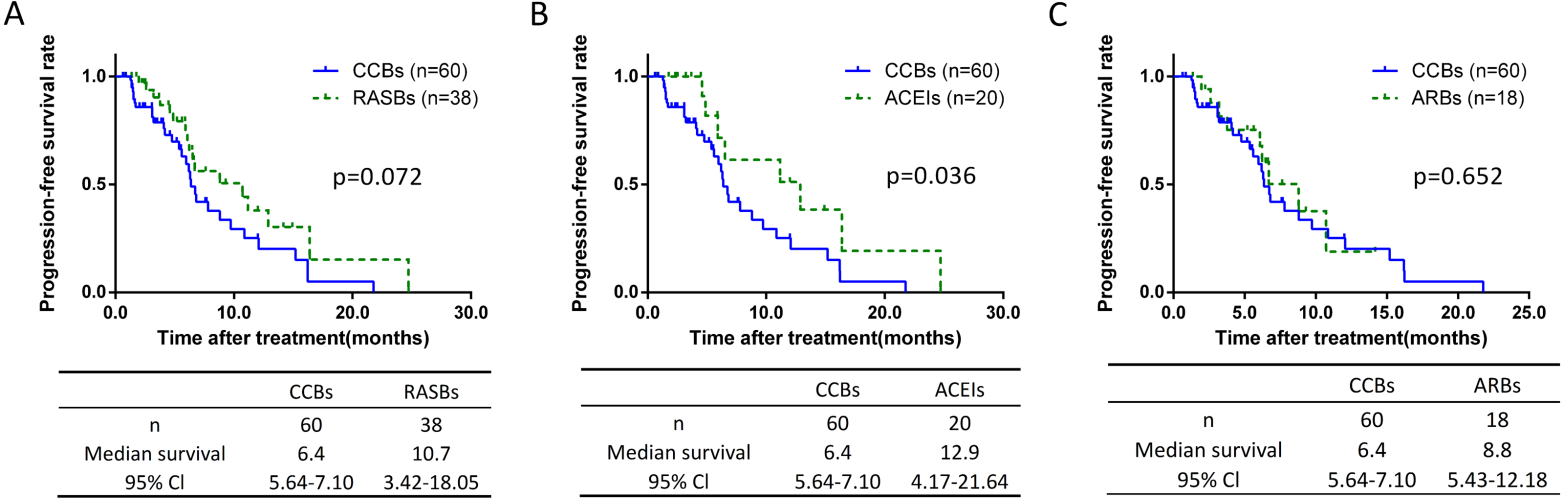
Kaplan-Meier curves for PFS of the CCBs group. Kaplan-Meier method analysis of PFS between (A) the CCBs group and RASBs group; (B) the CCBs group and ACEIs group; (C) the CCBs group and ARBs group. The statistical significance for difference of means is shown.

The non-RASB group included patients taking different types of antihypertensive drugs which are associated with an increased or decreased risk of lung cancer, and may have an impact. According to the antihypertensive drug class, we classified the non-RASB group, and found 77.9% (*n* = 359) of these cases were CCBs patients (Table 3). Further analysis showed there was no significant differences of lymph node metastasis and pathological stage between CCBs and the other groups (Table S1). The PFS of each group were assessed. Compared to the CCBs group, the PFS was 6.5 months longer in the ACEIs group with significantly difference (6.4 vs 12.9 months, *p* = 0.036) (Fig. 6B). However, no such difference was found between CCBs and RASBs groups (Fig. 6A), as well as CCBs and ARBs groups (Fig. 6C).

**Table 3:** Number of patients in the non-RASBs group receiving antihypertensive drugs.

Antihypertensive agent	Patients, *n*(%)
CCBs	359 (77.9)
Levamlodipine	149 (32.3)
Nitrendipine	69 (15.0)
Amlodipine	61 (13.2)
Nifedipine	57 (12.4)
Felodipine	13 (2.8)
Laxidipine	4 (0.9)
Lekadipine	2 (0.4)
Benidipine	1 (0.3)
Kalodipine	1 (0.3)
Nimodipine	1 (0.3)
Diuretics	30 (6.5)
Beta-blockers	11 (2.4)
Drug combination	37 (8.0)
Other drugs	24 (5.2)

Furthermore, we analyzed the prognostic significance of RASBs in adenocarcinoma and squamous cell carcinoma subgroups (Fig. S1), while the results showed no significant difference by relatively small sample size. The previous substantial epidemiologic evidence suggests that aspirin, which suppresses inflammation, reduces the risk of cancer (Gilligan et al., 2019). Therefore, we re-analyze lymph node metastasis, pathological stage (Table S2) and PFS (Fig. S2) after excluding patients with aspirin to minimize the bias. There is no statistical difference in lymph node metastasis and pathological stage. Consisting with former results in PFS. Compared with the non-RASBs group, the PFS of the RASBs and ACEIs groups was longer with significant difference (6.4 vs 12.9 months, *p* = 0.026), and the ARBs group did not.

## Discussion

Lung cancer, of which the main histological types are non-small cell lung cancer (NSCLC) and small cell lung cancer (SCLC), remains the most frequent cause of cancer-related mortality worldwide (Mei et al., 2018; Shi et al., 2017; Yan et al., 2018). Although substantive progress has been made in the diagnosis and targeted therapy of lung cancer, the impact of RASBs associated with metastasis and prognosis of lung cancer patients is still fairly unclear. In this study, for the first time, we showed that lung cancer patients who use RASBs and ARBs have reduced lymph node metastasis relative to non-RASBs. Lung cancer patients with RASBs have a lower pathological stage compared to those with non-RASBs, while the results of ACEIs refuted this finding. Similar results were revealed in cancer progression analysis. Contrasted with the non-RASBs group, RASBs and ACEIs have a preferable PFS of lung cancer, more specifically, in advanced pathological stage patients.

Lymph node metastasis in lung cancer, especially pathological status, is extremely important, not only for prognosis but also to direct postoperative therapeutic strategy (Yu et al., 2018). Previous studies have demonstrated that insertion/deletion polymorphisms of angiotensin-converting enzyme gene upregulate the risk of lymph node metastasis in human tumors such as laryngeal cancer and colorectal cancer (Han & Ge, 2016; Zheng et al., 2017). Recently, clinical data on the benefit of RASBs in primary and metastatic tumors proposed that, by activating immunostimulatory pathways, RASBs can enhance the immunotherapy of cancer and reduce or even prevent adverse effects associated with these therapies(Pinter & Jain, 2017). The anti-cancer effects of RASBs that inhibit cancer cell proliferation, tumor growth and metastasis were reported due to their immunoregulatory, antiangiogenic and anti-inflammatory mechanisms (Chen et al., 2013; Regulska, Stanisz & Regulski, 2013). Ethnic differences have been demonstrated to be an essential parameter related to ARBs and lung cancer risk, made evident by the results of a meta- analysis that showed that incidence of lung cancer was variable between Asians and Caucasians (Zhang et al., 2015). Furthermore, RASBs have already been proven to work as anti-lung cancer effectors with a prolonged PFS and overall survival in Chinese population (Cui et al., 2019; Miao et al., 2016; Wang et al., 2013), while no positive relation was found between lymph node metastasis and ACEIs/ARBs in Chinese populations (Wang et al., 2015). Thus, according to our study, the anti-hypertensive medication history of Chinese lung cancer patients could represent a much more convenient and reliable strategy for predicting whether lymph node metastasis during chemotherapy.

Previous studies showed that RASBs potentially have protective effects against lung cancer and taking RASBs can improve patient survival and mirror the positive effects of RASBs in combination with chemotherapy on PFS, both in the early and late stages of lung cancer, which was consistent with our study. However, Buchler et al. reported that ACEIs use among patients with multiple myeloma would lead to a shorter PFS (Buchler et al., 2005), which suggests that other molecular markers such as oncoproteins, miRNAs and lncRNAs may play an important role in prognosis and should be considered as one of the parameters calculated in multifactor models in future analyses. During anticancer radiotherapy and chemotherapy, treatment with DNA damage or targeted medicine may be associated with different dermatological adverse reactions. Radiation pneumonitis is a common problem of radiation-induced injury that significantly decreases patients’ quality of life and limits the therapeutic effect of radiation treatment. Although recent research has found that the risk of radiation-induced pneumonitis is significantly reduced in ACEIs cases, ARBs show the opposed effect (Wang et al., 2015). In this study, a difference between ACEIs and ARBs was not observed in chemotherapy patients. The possible reason may be a limited sample size and study design, such as a retrospective, nonrandom, and single-center trial. Thus, further research is needed to determine whether ACEIs and ARBs treatment cause pharmacodynamic differences in lung cancer and thus affect clinical outcomes and response to treatment.

There are several limitations in this retrospective single center study. We have analyzed several potential confounding factors such as individual characteristics, therapeutic protocols, and certain drugs. However, bias may be caused for instance to difference in number of antihypertensive drugs used, body mass index, concomitant disease and blood pressure control, etc, which are mainly related to lung cancer features. As the limitation of our database, we unable to assess BMI, comorbid conditions, and the changes of blood pressure in all included patients. Through previous studies have shown diabetes and cardiovascular disease could not influence prognosis in patients received RASBs (Menter et al., 2017), Lindgren et al. found that the risk increased by 10% for per 10 mmHg increment in systolic and diastolic blood pressure in lung cancer patients (Lindgren et al., 2003). All these finding suggested more attention should be paid about these unsure parameters in future prospective studies.

## Conclusions

In summary, for Chinese patients, the use of RASBs is a positive prognostic factor for clinical treatment including pathological stage, lymph node metastasis and patients’ PFS. Our study revealed that the use of RASBs can be a promising treatment option for lung cancer patients especially those receiving chemotherapy. The prognostic effects of RASBs should be validated in further prospective studies with larger datasets to evaluate the duration, timing, or type of RASBs and their influence on survival.

##  Supplemental Information

10.7717/peerj.8188/supp-1 Figure S1 Kaplan-Meier curves for PFS of Lung adenocarcinoma (A–C) or Lung squamous cell carcinoma (D–F)

10.7717/peerj.8188/supp-2 Figure S2 Kaplan-Meier curves for PFS after excluding aspirin Kaplan-Meier method to analyze PFS between (A) the non-RASBs group and RASBs group; (B) the non-RASBs group and ACEIs group; (C) the non-RASBs group and ARBs group. The statistical significance for difference of means is shown.

10.7717/peerj.8188/supp-3 File S1 The raw measurements of figures

10.7717/peerj.8188/supp-4 Table S1 Lymph node metastasis and pathological stage of CCB group CCBs calcium channel blockers; RASBs renin-angiotensin system blockers; ACEIs angiotensin-converting enzyme inhibitors; ARBs angiotensin-2 receptor 1 blockers; ^*a*^ Patients who took ACEIs and ARBs were excluded; ^#^ Each group was separately compared with the Non-RASBs group.

10.7717/peerj.8188/supp-5 Table S2 Comparison lymph node metastasis and pathological stage after excluding aspirin taken RASBs renin-angiotensin system blockers; ACEIs angiotensin-converting enzyme inhibitors; ARBs angiotensin-2 receptor 1 blockers; ^*a*^ Patients who took ACEIs and ARBs were excluded; ^#^ Each group was separately compared with the Non-RASBs group.

## Additional Information and Declarations

### Data Availability

The following information was supplied regarding data availability: The raw measurements of figures are available in the Supplementary Files.
