# Supplementary figures and images for: Retrospective clinical study of renin-angiotensin system blockers in lung cancer patients with hypertension
Source: PeerJ. 2019 Dec 10;7:e8188. doi: 10.7717/peerj.8188 (PMC6910116; doi:10.7717/peerj.8188)

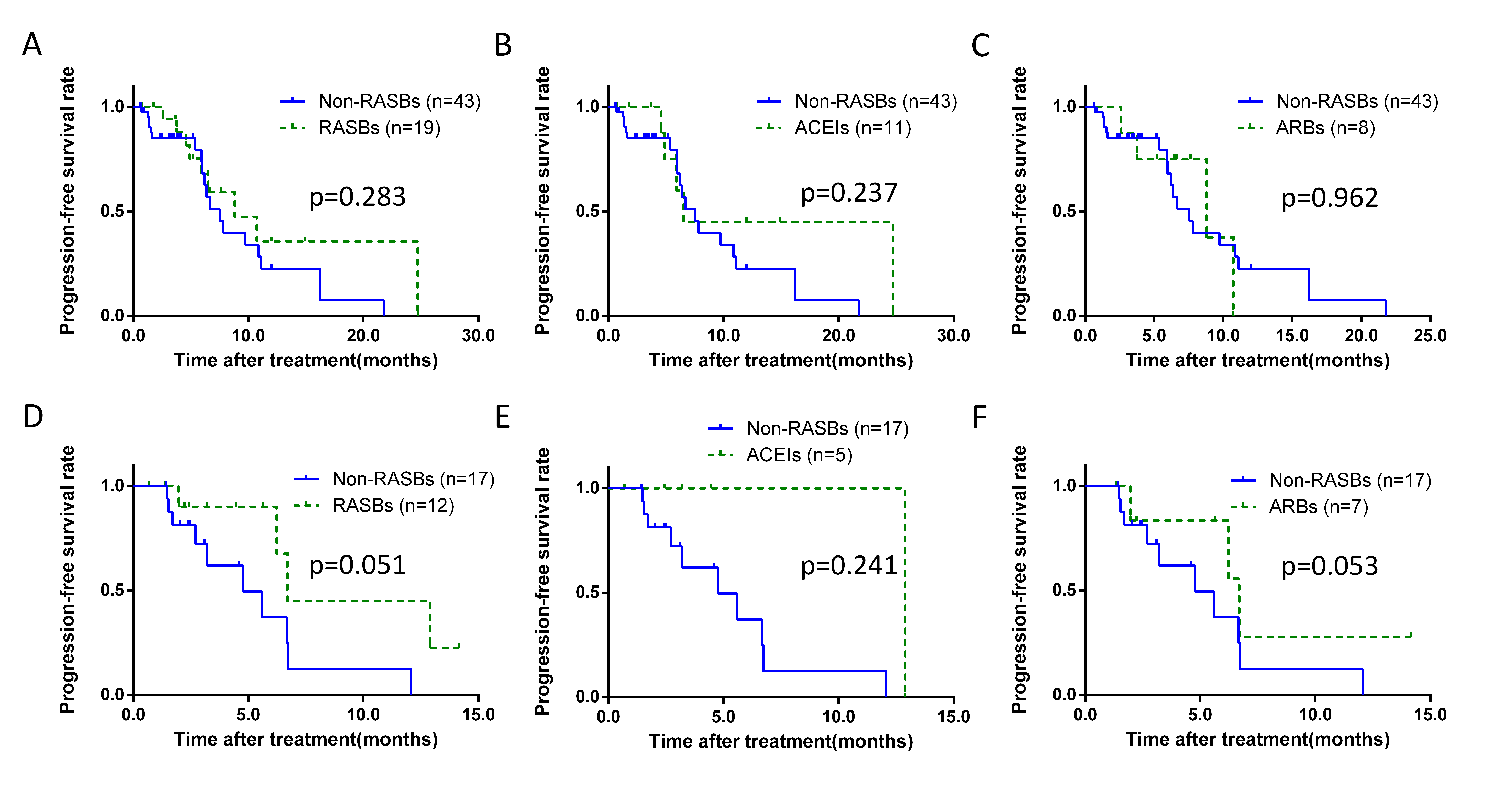

Supplement: Figure S1 [file peerj-07-8188-s001.png]

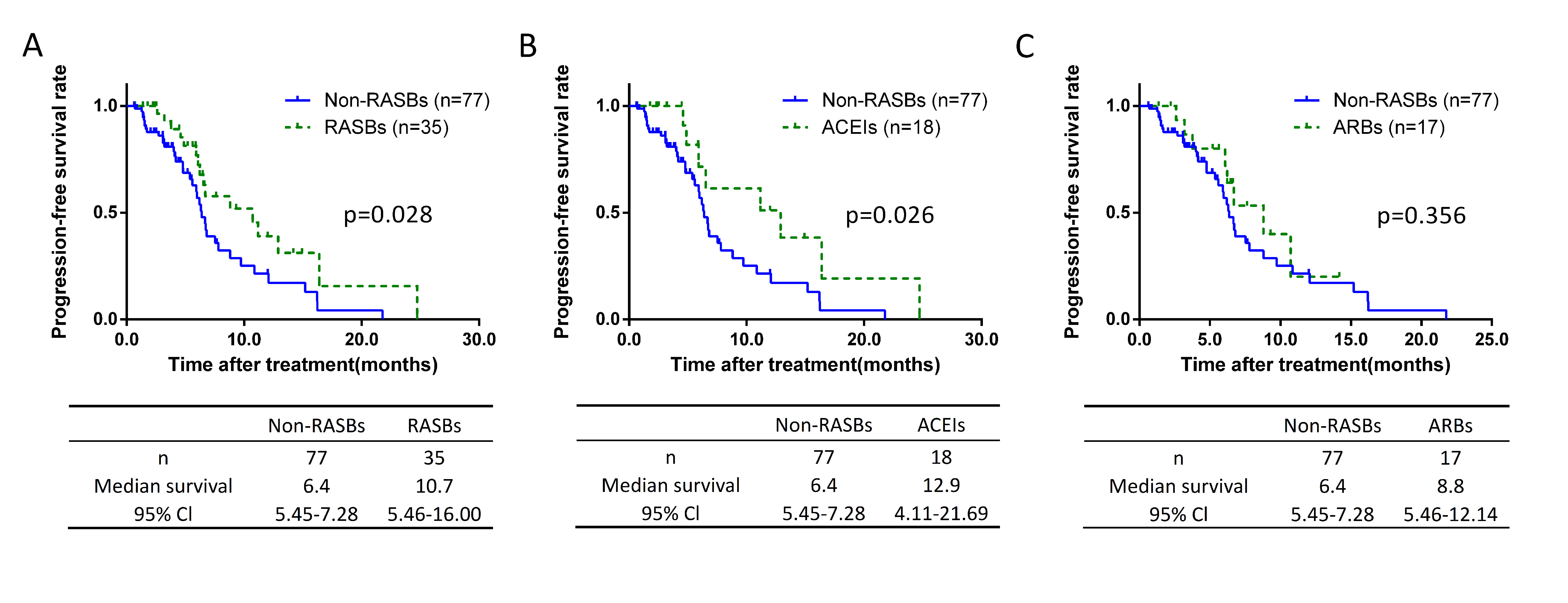

Supplement: Figure S2 — Kaplan-Meier method to analyze PFS between (A) the non-RASBs group and RASBs group; (B) the non-RASBs group and ACEIs group; (C) the non-RASBs group and ARBs group. The statistical significance for difference of means is shown. [file peerj-07-8188-s002.png]
