# Supplementary material for: Retrospective clinical study of renin-angiotensin system blockers in lung cancer patients with hypertension
Source: PeerJ. 2019 Dec 10;7:e8188. doi: 10.7717/peerj.8188 (PMC6910116; doi:10.7717/peerj.8188)
Supplement: Table S1 — CCBs calcium channel blockers; RASBs renin-angiotensin system blockers; ACEIs angiotensin-converting enzyme inhibitors; ARBs angiotensin-2 receptor 1 blockers; a Patients who took ACEIs and ARBs were excluded; # Each group was separately compared with the Non-RASBs group. [file peerj-07-8188-s004.doc]

| Characteristic | CCBs, n=359 | RASBs | | | | | |
| --- | --- | --- | --- | --- | --- | --- | --- |
|  |  | Total, n=201 | *P*-value^#^ | ACEIs ^a^, n=92 | *P*-value^#^ | ARBs ^a^, n=106 | *P*-value^#^ |
| Lymph node metastasis, n (%) |  |  |  |  |  |  |  |
| Yes | 207 (57.7) | 109 (50.2) | 0.093 | 53 (54.6) | 0.513 | 55 (47) | 0.081 |
| No | 85 (23.7) | 63 (29) |  | 26 (26.8) |  | 35 (29.9) |  |
| Unknown | 67 (18.6) | 45 (20.8) |  | 18 (18.6) |  | 27 (23.1) |  |
| Pathological stage, n (%) |  |  |  |  |  |  |  |
| I | 150 (41.8) | 75 (34.6) | 0.083 | 28 (28.9) | 0.066 | 46 (39.3) | 0.213 |
| II | 66 (18.4) | 41 (18.9) |  | 24 (24.7) |  | 17 (14.5) |  |
| III | 25 (6.9) | 22 (10.1) |  | 11 (11.3) |  | 11 (9.4) |  |
| IV | 38 (10.6) | 35 (16.1) |  | 13 (13.4) |  | 20 (17.1) |  |
| Unknown | 80 (22.3) | 44 (20.3) |  | 21 (21.7) |  | 23 (19.7) |  |
