# Supplementary material for: Retrospective clinical study of renin-angiotensin system blockers in lung cancer patients with hypertension
Source: PeerJ. 2019 Dec 10;7:e8188. doi: 10.7717/peerj.8188 (PMC6910116; doi:10.7717/peerj.8188)
Supplement: Table S2 — RASBs renin-angiotensin system blockers; ACEIs angiotensin-converting enzyme inhibitors; ARBs angiotensin-2 receptor 1 blockers; a Patients who took ACEIs and ARBs were excluded; # Each group was separately compared with the Non-RASBs group. [file peerj-07-8188-s005.doc]

| Characteristic | Non-RASBs, n=443 | RASBs | | | | | |
| --- | --- | --- | --- | --- | --- | --- | --- |
|  |  | Total, n=201 | *P*-value^#^ | ACEIs ^a^, n=92 | *P*-value^#^ | ARBs ^a^, n=106 | *P*-value^#^ |
| Lymph node metastasis, n (%) |  |  |  |  |  |  |  |
| Yes | 259 (58.5) | 103 (51.2) | 0.101 | 51 (55.4) | 0.538 | 51 (48.1) | 0.096 |
| No | 103 (23.2) | 57 (28.4) |  | 24 (26.1) |  | 31 (29.3) |  |
| Unknown | 81 (18.3) | 41 (20.4) |  | 17 (18.5) |  | 24 (22.6) |  |
| Pathological stage, n (%) |  |  |  |  |  |  |  |
| I | 189 (42.7) | 69 (34.3) | 0.059 | 28 (30.4) | 0.050 | 40 (37.7) | 0.170 |
| II | 76 (17.1) | 38 (18.9) |  | 23 (25) |  | 15 (14.2) |  |
| III | 27 (6.1) | 20 (10) |  | 10 (10.9) |  | 10 (9.4) |  |
| IV | 51 (11.5) | 32 (15.9) |  | 11 (12) |  | 19 (17.9) |  |
| Unknown | 100 (22.6) | 42 (20.9) |  | 20 (21.7) |  | 22 (20.8) |  |
